# In this issue: Volume 115, Issue 8, August 2024

**DOI:** 10.1111/cas.16293

**Published:** 2024-08-08

## Cutibacterium acnes‐derived extracellular vesicles promote tumor growth in renal cell carcinoma

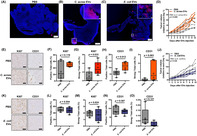

The human body harbors bacterial flora in various locations, including the intestine, and are involved in various diseases, like diabetes, chronic kidney diseases, and intestinal diseases. Renal cell carcinoma (RCC) is a type of kidney cancer influenced by the body's metabolic environment, including gut bacteria. Recent discoveries have found bacterial DNA in the bloodstream, which was once thought to be a sterile environment, suggesting it could be used as a diagnostic tool for cancer. It has been known that tumors have their own bacterial flora, but the impact of these bacteria on tumor growth and whether blood and tumor bacterial profiles match remain unclear.

In this study, Jingushi et al. investigated the role of bacteria in RCC progression. They focused on extracellular vesicles (EVs), which are tiny particles carrying bacterial DNA, by performing 16S rRNA metagenomic analysis on blood samples taken from seven patients with RCC and five healthy donors. The analysis revealed that serum EVs from patients with RCC contained the distinctive bacterial DNA of *Cutibacterium acnes*. Furthermore, compared to preoperative serum EVs from patients with RCC, postoperative serum EVs from these patients had significantly less *C. acnes* DNA. The study also showed that *C. acnes* DNA was found in tumor tissue and associated EVs, indicating a possible link between these bacterial vesicles and RCC.

To explore whether *C. acnes* promotes tumor growth in vivo, the researchers administered *C. acnes* EVs in a mouse model of renal allografts. They observed that *C. acnes* EVs enhanced tumor growth in vivo with formation of new blood vessels.

In conclusion, the study showed that EVs released by *C. acnes* were taken up by cancer cells, promoting tumor growth. The findings highlight the potential role of bacterial EVs in cancer progression and could lead to new diagnostic and therapeutic strategies for RCC.

https://onlinelibrary.wiley.com/doi/10.1111/cas.16202

## Alternative magnetic field exposure suppresses tumor growth via metabolic reprogramming

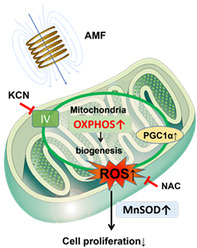

Current cancer treatments involve a range of physical forces, such as ultrasound and electric fields. Despite their widespread use, the exact mechanisms by which these forces affect cancer cells remain unclear. This study explores the effects of an alternating magnetic field (AMF) on glioblastoma multiforme (GBM), a highly aggressive brain tumor.

In this study, Akimoto et al. investigated the impact of AMF exposure on GBM cells. They found that a specific narrow AMF frequency range, including around 227 kHz, significantly inhibits the growth of these cells. This inhibition is linked to an increase in oxidative phosphorylation within the cancer cells, leading to metabolic reprogramming.

In the cell experiments, AMF exposure slowed the growth of GBM cells but did not affect non‐cancerous cells, such as normal human astrocytes, a type of brain cells. The researchers then tested this treatment in mice implanted with human GBM cells. Daily AMF treatment for 30 min over 3 weeks reduced tumor growth and extended survival times in the test animals. This treatment was shown to be linked with enhanced levels of reactive oxygen species (ROS) and manganese superoxide dismutase, which impaired cancer cell metabolism and growth. Unsurprisingly, the effects of the treatment were reduced by a mitochondrial complex IV inhibitor or a ROS scavenger; this finding, along with the observed decrease in extracellular acidification rate and increase in oxygen consumption rate, strongly attests to the likelihood of AMF targeting cancer cells only.

One significant advantage of the AMF therapy is its non‐invasive nature. Unlike electric fields that require direct contact with tissues, AMF can penetrate deep into the body without contact, making it less invasive. The therapy's non‐contact nature and short treatment duration make it practical for clinical use.

This study highlights the potential of using magnetic fields to disrupt cancer cell metabolism and reduce tumor growth effectively and safely. AMF promises to be a potential new tool in cancer treatment. Further research is needed to understand the full clinical implications of AMF therapy for cancer patients.

https://onlinelibrary.wiley.com/doi/full/10.1111/cas.16243

## Single‐cell landscape reveals the immune heterogeneity of bone marrow involvement in peripheral T‐cell lymphoma

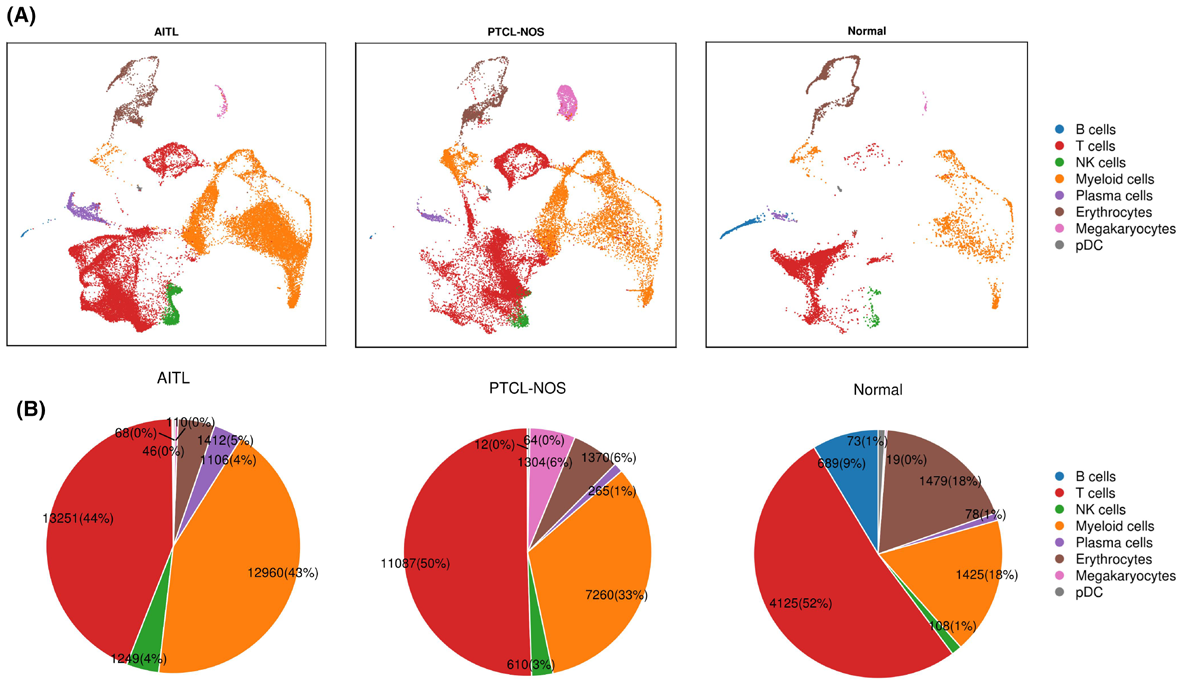

Peripheral T‐cell lymphoma (PTCL) is a serious type of blood cancer that affects the immune T‐cells. The outlook for PTCL patients heavily depends on whether it spreads to the bone marrow (BM). However, understanding the BM environment, specifically in terms of the immune cells and genetic variations, in PTCL has been challenging.

To bridge this gap, Liu et al. investigated the complexity of the BM microenvironment using single‐cell RNA sequencing. The researchers used BM samples from 11 PTCL patients whose cancer had spread to the BM microenvironment for the thorough analysis of the genetic activities of the individual cells.

The findings underscored clear differences between two types of PTCL: angioimmunoblastic T‐cell lymphoma (AITL) and PTCL not otherwise specified (NOS). AITL had more T‐cells, fewer lymphocytes, and higher level of inflammation when compared to PTCL‐NOS. These differences in AITL were linked to patient prognosis and the superior response of patients with a higher number of a certain T‐cell type to a specific therapy called anti‐CD30 therapy.

In these patients, the researchers also identified new genetic markers related to RhoA mutation, which could potentially serve as future therapeutic targets. Importantly, they found that patients treated with the drug chidamide had more CD4+ regulatory cells in their BM, which improved their response to treatment. However, patients who did not respond well to the drug had an increase in immune B‐cells and eventually developed a different type of lymphoma.

Additionally, Liu et al. found that AITL patients with a condition called lymphoma‐associated hemophagocytic syndrome had more T follicular helper cells with genetic variations in chromosome 5. The finding suggests that genetic differences can play a crucial role in how the disease progresses and how patients respond to treatment.

Overall, the study suggests that therapies like brentuximab vedotin and chidamide might modify immune responses in PTCL patients, allowing for tailored treatment plans based on individual immune and genetic traits. This insight paves the way for personalized PTCL therapies that go beyond general approaches, offering better outcomes and renewed optimism for individuals confronted with this challenging disease.

https://onlinelibrary.wiley.com/doi/full/10.1111/cas.16227

